# Chromatin remodeling during the *in vivo* glial differentiation in early *Drosophila* embryos

**DOI:** 10.1038/srep33422

**Published:** 2016-09-16

**Authors:** Youqiong Ye, Liang Gu, Xiaolong Chen, Jiejun Shi, Xiaobai Zhang, Cizhong Jiang

**Affiliations:** 1 Department of Clinical Laboratory Medicine, Shanghai Tenth People’s Hospital of Tongji University, Shanghai Key Laboratory of Signaling and Disease Research, the School of Life Sciences and Technology, the Collaborative Innovation Center for Brain Science, Tongji University, Shanghai 200092, China

## Abstract

Chromatin remodeling plays a critical role in gene regulation and impacts many biological processes. However, little is known about the relationship between chromatin remodeling dynamics and *in vivo* cell lineage commitment. Here, we reveal the patterns of histone modification change and nucleosome positioning dynamics and their epigenetic regulatory roles during the *in vivo* glial differentiation in early *Drosophila* embryos. The genome-wide average H3K9ac signals in promoter regions are decreased in the glial cells compared to the neural progenitor cells. However, H3K9ac signals are increased in a group of genes that are up-regulated in glial cells and involved in gliogenesis. There occurs extensive nucleosome remodeling including shift, loss, and gain. Nucleosome depletion regions (NDRs) form in both promoters and enhancers. As a result, the associated genes are up-regulated. Intriguingly, NDRs form in two fashions: nucleosome shift and eviction. Moreover, the mode of NDR formation is independent of the original chromatin state of enhancers in the neural progenitor cells.

Epigenetic factors play a critical role in many biological processes through regulating gene transcription. Chromatin remodeling is one of the key epigenetic mechanisms, mainly including nucleosome positioning and histone modification dynamics. Nucleosome is the fundamental repeating structural unit of chromatin in Eukaryotes. It regulates DNA template-based processes, such as DNA replication, DNA repair, and transcription, by controlling DNA accessibility^1^. Recent findings have indicated an open chromatin state in pluripotency stems cells while a relatively condensed chromatin structure in lineage-committed cells^2^. Nucleosome depletion occurred in the upstream regions proximal to transcription start sites (TSSs) of the genes activated during undifferentiated mouse embryonic stem cells (ESC) E14 differentiating to endoderm/hepatic progenitor (EHP) cells^3^. Comparison of genome-wide nucleosome occupancy in mouse ESCs, the neural progenitor cells, and embryonic fibroblasts revealed important roles of nucleosome positioning in cell differentiation^4^. A recent study also found that regions of difference in nucleosome occupancy were enriched in genes and regulatory elements during cell differentiation and reprogramming^5^.

There are a multiplicity of chemical modifications on histone tails of nucleosomes. Histone modifications function either by changing chromosomal state or by recruiting nonhistone proteins to chromatin^6^. As a result, histone modifications influence many fundamental biological processes. A previous study showed different change patterns of H3K9 acetylation, H3K9 and H3K4 methylation, and global histone deacetylation during mouse ESCs differentiation^7^. The study on histone modification dynamics during hematopoietic differentiation revealed that the *de novo* established enhancers can predict the differentiation potential of progeny^8^. A comprehensive analysis of mouse ESCs and neural progenitor cells identified dozens of core histone modification sites in the two cell types, respectively, and elucidated the effect of combinatorial histone modifications in the differentiation^9^. Interestingly, nucleosome occupancy was correlated with different histone modifications during the differentiation of mouse ESCs to neural progenitor cells^4^. However, all these studies used homogeneous cell culture or *in vitro* differentiated cells and failed to represent the scenarios under the proper *in vivo* context within the organism.

Glia is a major cell type of the nervous system and known as “supporting cells” that provide support and protection for neurons. Glial cells differentiate from multipotent neural stem cells. In *Drosophila* early embryos, a single transcription factor Glial Cells missing (Gcm) drives glial differentiation from the multipotent neural progenitor cells^10^. *Gcm* is transiently expressed in these progenitor cells and can be used as a marker gene^11^. One of Gcm target genes, the reverse polarity (*Repo*), is specifically expressed in glial cells^12^,^13^. Together with relative simple *Drosophila* central nervous system, it is feasible to explore the chromatin remodeling and its role during the *in vivo* glial differentiation in *Drosophila* embryos.

In this study, we employ INTACT (isolation of nuclei tagged in specific cell types) approach^14^ to explore the chromatin remodeling during glial differentiation under the proper *in vivo* context. INTACT-captured cell-type specific nuclei can be used for gene expression, epigenomic, and proteomic profiling^15^,^16^,^17^. Our approach uses the Gal4-UAS system to express a tagged nuclear membrane protein specifically in the multipotent neural progenitor cells under the control of *Gcm* promoter and in glial cells under the control of *Repo* promoter, respectively. Thus, we isolated the multipotent neural progenitor cells and glial cells by affinity purification from early *Drosophila* embryos. We profiled gene expression and chromatin state (nucleosome occupancy and core histone modifications) of the two cell types through high-throughput sequencing technology. Our results reveal different change patterns of core histone modifications in promoters and extensive nucleosome remodeling. Increased H3K9ac signals up-regulate the group of genes important to gliogenesis. NDRs form in both promoters and enhancers. Moreover, the mode of NDR formation is independent of the chromatin state of enhancers in the neural progenitor cells.

## Results

### High purity of affinity-isolated nuclei for the two cell types

**Figure 1:**
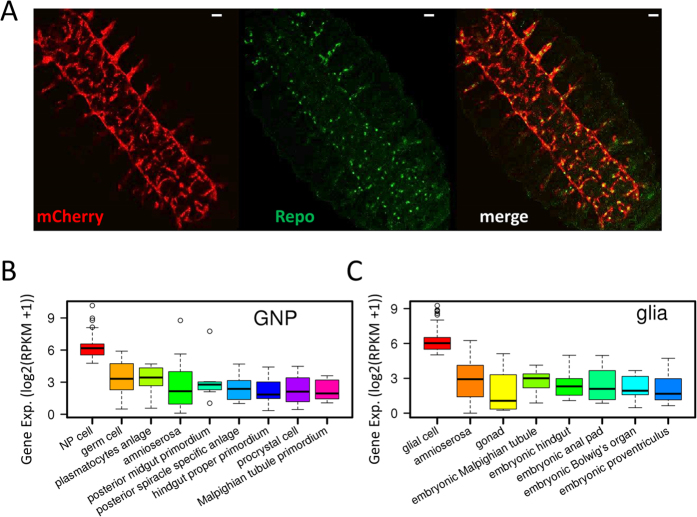
Affinity purification of glial nuclei from *Drosophila* embryos. (**A**) Detection of mCherry (red) epitope and endogenous Repo (green) expression in a fixed stage-15 embryo (*Repo*-*Gal4* > *UAS*-*NTF*). Scale bar: 20 μm. (**B**) The expression levels of neural progenitor (NP) cell-specific genes are significantly higher than other tissue-specific genes in the isolated nuclei of GNP cells. (**C**) The expression levels of glial cell-specific genes are significantly higher than other tissue-specific genes in the isolated glial nuclei. All p-values are less than 0.01 in (**B**,**C**) (Wilcoxon rank sum test).

We first generated *Drosophila* line containing *Gcm-Gal4* > *UAS-NTF (Gcm* > *NTF*) for affinity purification of the multipotent neural progenitor cells (referred to as GNP hereafter, *Gcm*-expressed Neural Progenitor). The *NTF* gene is the nuclear targeting fusion gene introduced in INTACT approach^17^ that consists of 3xFLAG, BLRP (biotin ligase recognition peptide, a preferred substrate for BirA), mCherry, and RanGap (expressed in the cytoplasm and outer nuclear envelope). The nuclei of GNP cells were purified by affinity from stage 11 embryos (5–7h AEL) using anti-Flag-coated magnetic beads. Similarly, we created *Drosophila* line containing *Repo*-*Gal4* > *UAS*-*NTF (Repo* > *NTF*) for affinity purification of glial nuclei from stage 15–16 embryos (12–14h AEL) (Fig. 1A). The purity of isolated GNP and glial nuclei reached 92.4% and 99.5%, respectively (Supplementary Fig. S1A). That is, 92.4% of affinity-purified GNP nuclei express *Gcm* and 99.5% of isolated glial nuclei express *Repo*.

We next collected different tissue-specific genes that were determined by RNA *in situ* hybridization from Berkeley *Drosophila* Genome Project^18^ and profiled the gene expression. The results show that expression levels of neural progenitor (NP) cell-specific genes are significantly higher than other tissue-specific genes in GNP cells. Similarly, expression levels of glial cell-specific genes are significantly higher than other tissue-specific genes in glial cells (Fig. 1B,C). Consistently, the active histone modification contents (H3K4me3 and H3K9ac) in promoter regions of NP cell-specific genes are significantly higher than other tissue-specific genes in GNP cells. We observed the same pattern of the histone modification signals for glial cell-specific genes in glial cells (Supplementary Fig. S1B,C). These findings demonstrate that expression profiles are consistent with the cell identities and suggest that the purified nuclei suitable for expression and chromatin profiling.

### Gene expression changes during the glial differentiation

**Figure 2:**
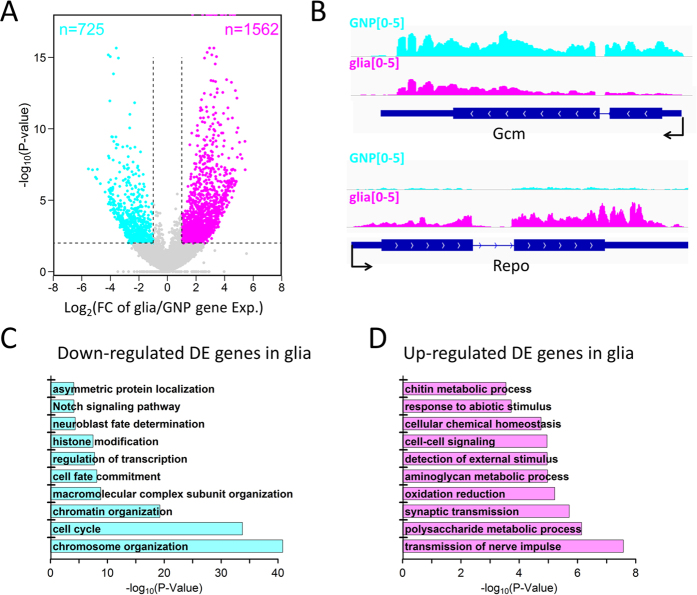
Expression profiles during the glial differentiation. (**A**) Volcano plot shows the significantly differentially expressed (DE) genes in colors, up-regulated (magenta) and down-regulated (cyan) in glia. The numbers of the DE genes are also given. (**B**) Track view for expression levels of the two marker genes *Gcm* and *Repo* in the two cell types. (**C,D**) GO analysis results for DE genes, down-regulated (**C**) and up-regulated (**D**) in glia.

Gene expression changes can provide cues for understanding the molecular mechanisms of biological processes. Therefore, we identified significantly differentially expressed (DE) genes during the glial differentiation, 725 down-regulated and 1562 up-regulated in glia (Fig. 2A). For example, the glial marker gene *Repo* is significantly up-regulated during the glial differentiation whereas the GNP marker gene *Gcm* is down-regulated (Fig. 2B). GO term analysis of the down-regulated DE genes identified enrichment for cell cycle, cell fate commitment, neuroblast fate determination, Notch signaling pathway, etc. stem cell-related functions (Fig. 2C). In contrast, the up-regulated DE genes are enriched for glial function-related GO terms such as transmission of nerve impulse, synaptic transmission, etc. (Fig. 2D). This implies that the gene expression program is switched to meet the distinct functions of these two neural cell types during the glial differentiation.

### Chromatin remodeling in the promoter regions

Chromatin state in the promoter regions plays a critical role in gene activity. Profiles of the core histone modification signals in the promoter regions show three major chromatin states: H3K4me3+/H3K9ac+, H3K27me3+, and none (Supplementary Fig. S2A). There are only few bivalent promoters (H3K4me3+/H3K27me3+). This is consistent with the previous finding that bivalency is not prevalent in fly embryo epigenome^19^. In order to reveal the impact of chromatin state on gene activity, we profiled expression levels of genes grouped by the chromatin state in the promoters. The results show that the expression levels of genes marked by H3K4me3+/H3K9ac+ are significantly higher than other groups of genes. The genes marked by H3K27me3+ have the lowest expression levels (Supplementary Fig. S2B). This indicates that chromatin states in the promoter regions are a good predictive for gene activity.

**Figure 3:**
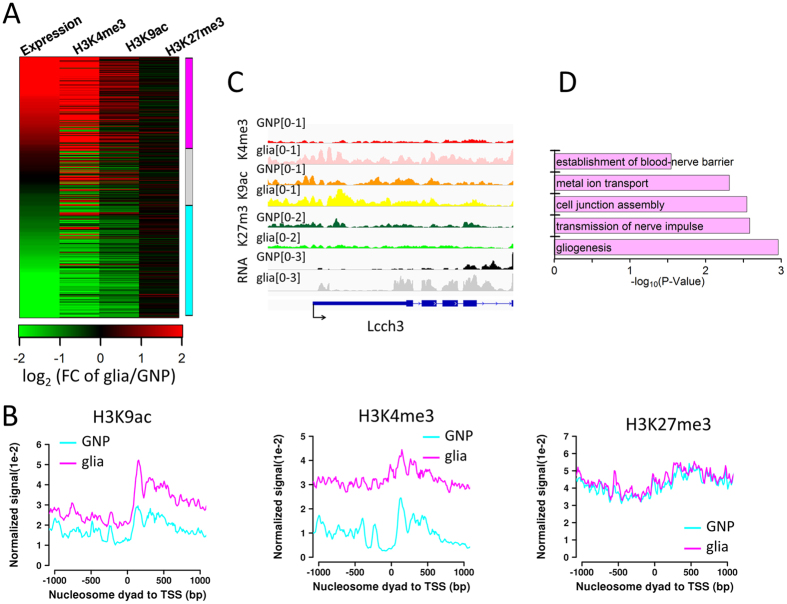
Histone modification change in promoter regions. (**A**) Heatmap showing fold change of expression levels and core histone modification signals of the collected glia-related genes. The genes are ordered by the fold change of gene expression levels and divided into three groups: up-regulated (>1.5 fold, magenta), no change (gray), and down-regulated (>1.5 fold, cyan) in glia. (**B**) Core histone modification signals in the regions around TSS of the up-regulated genes in (**A**). (**C**) Track view for expression levels, core histone modification signals in the glial gene *Lcch3* In the two cell types. (**D**) GO analysis results for the up-regulated genes in (**A**).

We further investigated how chromatin state dynamics functions in the glial differentiation. A total of 368 genes closely related to glial development and differentiation were collected from Berkeley *Drosophila* Genome Project^18^ and previous studies^20^,^21^. We correlated chromatin state dynamics in the promoters and the gene expression change, and found that the active histone marks H3K4me3 and H3K9ac positively regulated gene expression change whereas the repressive histone modification H3K27me3 had little effect on gene expression change (Fig. 3A). The expression levels are significantly increased and decreased in the designated two groups of genes: up-regulated and down-regulated, respectively (Supplementary Fig. S2C). A previous study found that low levels of histone acetylation was required for glial differentiation, especially, low levels of H3K9ac in endogenous glial cells^11^. Consistently, we observed that the overall H3K9ac signals in promoters was lower in glial cells than in GNP cells (Supplementary Fig. S2D). Here, it raises the question as whether it is the same case for the genes that function in glia and are up-regulated during the glial differentiation. To address this, we further examined the H3K9ac signal change in the promoters of such genes and surprisingly found that H3K9ac signals were increased in these genes during the glial differentiation (Fig. 3B,C and Supplementary Fig. S2E). This pattern is opposite to the overall pattern (Supplementary Fig. S2D). This finding is likely due to the higher resolution of ChIP-seq in this study compared to the immunolabeling in the previous study. Another active histone marker H3K4me3 signals are also increased in this group of genes. In contrast, There is no significant change in the repressive marker H3K27me3 (Fig. 3B). Interestingly, GO term analysis of this group of the up-regulated genes identified enrichment for glia-related GO terms such as gliogenesis, transmission of nerve impulse, etc. (Fig. 3D). Together, the results suggest that the average H3K9ac occupancy levels in the promoters decrease during the glial differentiation while H3K9ac occupancy levels increase in the up-regulated genes with glial functions.

### Nucleosome positioning dynamics in the promoter regions

To gain insights into nucleosome remodeling and its role during the glial differentiation, we first scanned the genome using a 200-bp window and calculated nucleosome occupancy in each window for the two cell types. Comparison analysis show that extensive nucleosome remodeling occurs during the glial differentiation and it results in a relative more closed chromatin structure (Supplementary Fig. S3A). This is consistent with the previous results that differentiated cells have a more condensed chromatin structure compared to pluripotent stem cells^2^.

**Figure 4:**
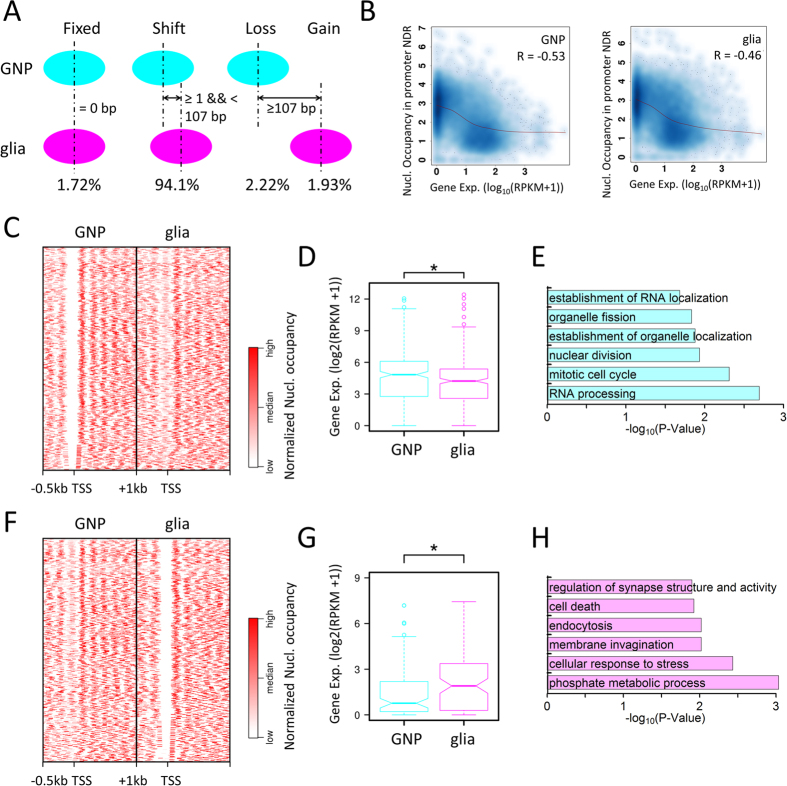
Nucleosome positioning dynamics in promoter regions. (**A**) Schematic for nucleosome remodeling: fixed nucleosome, nucleosome shift, loss, and gain. (**B**) Negative correlation between nucleosome occupancy in promoter NDRs and gene expression level. Spearman’s correlation coefficients are given. (**C**) Heatmap showing NDRs in the region [−200, +50 bp] of TSS in the GNP cells that are occupied by nucleosome(s) in the glial cells. **(D)** Expression profiles of the genes in (**C**) (*p < 0.05, Wilcoxon rank sum test). (**E**) GO analysis results for the genes in (**C**). (**F**) Heatmap showing NDRs in the region [−200, +50 bp] of TSS in the glial cells that are originally occupied by nucleosome(s) in the GNP cells. (**G**) Expression profiles of the genes in (**F**) (*p < 0.05, Wilcoxon rank sum test). (**H**) GO analysis results for the genes in (**F**).

We next predicted genome-wide nucleosome positions using the tool GeneTrack^22^ and analyzed nucleosome positioning changes on the basis of the distance between the two closest nucleosomes of the two cell types. Only 1.72% of nucleosomes remain the exactly same positions. 4.15% of nucleosomes are lost (2.22%) or gained (1.93%) during the glial differentiation. That is, the two closest nucleosomes from the two cell types overlap less than or equal to 20 bp. The rest of nucleosomes (94.1%) shift their positions (Fig. 4A). Nucleosome gain or loss is enriched in the promoter regions (Supplementary Fig. S3B). The previous study has revealed that NDRs in the promoter regions open chromatin and facilitate transcription^23^. Consistently, The nucleosome occupancy in NDRs in the promoter regions in GNP cells is significantly lower in NP cell-specific genes than other tissue-specific genes. Similarly, the nucleosome occupancy in NDRs in the promoter regions in glial cells is significantly lower in glial cell-specific genes than other tissue-specific genes (Supplementary Fig. S3C). Consistently, the NP cell-specific genes have higher expression levels in GNP cells than other tissue-specific genes. It is the same case for the glia-specific genes in the glial cells (Fig. 1B,C). Overall, we found nucleosome occupancy in NDRs in the promoter regions is negatively correlated with gene expression levels in GNP cells and glia (Fig. 4B). We further focused on the 545 genes that have an NDR in the promoter in GNP cells and the NDR is occupied by an nucleosome in glial cells. The expression levels of these genes are significantly higher in GNP cells than in glial cells. GO term analysis of these genes revealed that they were enriched for cell cycle, nuclear division, etc. functions related to stem cell pluripotency (Fig. 4C–E). Conversely, an NDR forms in the promoter regions of another set of genes (539) during the glial differentiation. Consequently, their expression levels are significantly increased in glial cells. These genes are enriched for glial function related GO terms: phosphate metabolic process, cell death, etc. (Fig. 4F–H).

### Nucleosome positioning dynamics in enhancers

Enhancers are distal *cis* regulatory elements beyond promoters and play a critical role in establishing and maintaining cell identity^24^,^25^. We identified 3698 enhancers and classified them by histone modifications in a similar way used in the previous study^25^. We designated the H3K4me1+/H3K27ac+/H3K27me3- enhancers as active, the H3K4me1+/H3K27ac-/H3K27me3+ enhancers as poised, and the rest as intermediate. Consistent with this classification, expression analyses reveal that the active enhancer associated genes are expressed at the highest levels, the poised enhancer associated genes are expressed at the lowest levels, and the intermediate enhancer associated genes are expressed at the intermediate levels (Supplementary Fig. S4A). Interestingly, 89.1% of poised enhancers in GNP cells remain poised in glial cells. GO term analyses of the genes associated with these enhancers identified enrichment for non-neural tissue development such as head segmentation, leg disc development, heart development, etc. (Supplementary Fig. S4B).

**Figure 5:**
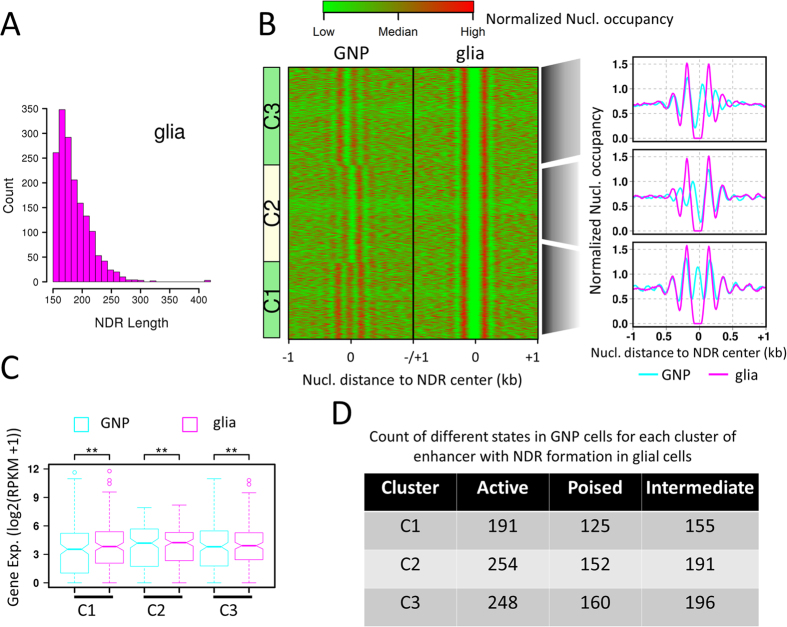
Nucleosome positioning dynamics in enhancers. (**A**) Frequency distribution of length of enhancer NDRs. (**B**) Heatmap: clustering view of nucleosome organization in ±1 kb regions centering at NDRs in glial enhancers. Curve plots: the composite distribution of nucleosomes to the center of NDRs in glial enhancers. (**C**) Expression profiles of the genes associated with the enhancers in (**B**). (**D**) Statistics of enhancers in (**B**) categorized by the chromatin state in the GNP cells.

Nucleosome remodeling in enhancers changes DNA accessibility and regulates gene activity. Thus, we scanned enhancers and identified regions of at least 150 bp (~length of a nucleosome). These regions are defined as enhancer NDRs. Their length ranges from 154–417 bp and peaks at 163 bp (Fig. 5A). This indicates that only one nucleosome is disassembled within enhancers in most cases during the glial differentiation. Total of 1672 NDRs form in glial enhancers. We clustered these enhancers based on their nucleosome occupancy in GNP cells and obtained three groups (Fig. 5B). Comparison of nucleosome occupancy in the regions centering these NDRs between GNP and glial cells shows that an nucleosome is evicted to form the NDRs in C1 group during glial differentiation. In contrast, the upstream nucleosomes shift upstream to form the NDRs in C2 group whereas the downstream nucleosomes shift downstream to form the NDRs in C3 group (Fig. 5B). Therefore, these NDRs in glial enhancers are generated in two modes: nucleosome eviction and shift. The expression levels of the genes associated with these enhancers are significantly increased in glial cells than in GNP cells (Fig. 5C). This suggests that NDR formation within enhancers facilitates gene transcription. We next examined the chromatin state of these enhancers in GNP cells. The results show that there is no significantly difference in the chromatin state distribution in GNP cells between enhancers with NDR formation through nucleosome eviction and shift. There is also no bias in either of the three chromatin states: active, poised, and intermediate (Fig. 5D). This finding indicates that NDR formation mode in the glial cells is independent of enhancer original chromatin state in the neural progenitor cells.

## Discussion

Chromatin structure is the underpinning of gene regulation. Nucleosome occupancy and post-translational histone modifications are the key two chromatin remodeling factors and play a critical role in cell differentiation. Profiling analyses of gene expression and core histone modification signals in affinity-purified neural progenitor cells and glial cells from early *Drosophila* embryos show a low level of average H3K9ac signals in promoters in glial cells compared to GNP cells. However, H3K9ac signals are still increased in the group of genes that are involved in glial functions and whose expression levels are increased during the glial differentiation. Nucleosome positioning facilitates the glial differentiation through NDR formation in both promoters and enhancers. NDRs form in enhancers in two modes: nucleosome shift and eviction. These findings shed new light on patterns of chromatin remodeling and its epigenetic regulatory role during *in vivo* neural progenitor cells differentiating into glial cells.

*Gcm* regulates both glial cell differentiation and the development of the plasmatocyte/macrophage lineage of hemocytes^26^,^27^,^28^. It starts expression in the embryo at stage 5 and continues expression in hemocyte precursors through stage 11^29^,^30^. This raises the question as to whether the affinity-purified GNP cells can represent NP cells. Expression profiling of different tissue specific gene sets in GNP cells shows that the expression levels of NP cell-specific genes are significantly higher than hemocyte lineage genes (plasmatocytes anlage) and other tissue specific genes (Fig. 1B). Consistently, H3K4me3 and H3K9ac signals in the promoters of NP cell specific genes are significantly higher than hemocyte lineage genes and other tissue specific genes in GNP cells (Supplementary Fig. S1B). This suggests that affinity-purified GNP cells indeed mainly contain NP cells. As a matter of fact, it was reported that *Gcm* expression rapidly disappears after stage 11^29^. Coincidentally, GNP cells are isolated from stage-11 embryos in this study. Taken together, affinity-purified GNP cells from stage-11 embryos are appropriate for profiling of gene expression and chromatin state for NP cells.

Histone modifications play an important role in cell fate determination. It has been reported that low levels of H3K9ac signals are a prerequisite for glial differentiation^11^. However, the technical limit of immunohistochemical staining failed to examine histone modification signals of a certain group of genes. In contrast, ChIP-seq technology used in this study allowed us to observe increased H3K9ac signals in a group of genes important to glial differentiation as well as the overall low H3K9ac levels.

Enhancers are distal *cis* regulatory elements and also play a critical role in lineage commitment^31^,^32^,^33^. The study on chromatin state dynamics during hematopoietic differentiation found that enhancer establishment was initiated earlier and could predicate the differentiation potential of progenitor cells prior to gene expression profiles^8^. Our results show that NDRs form in enhancers and regulate gene expression during the glial differentiation. It will be interesting to know whether NDRs within enhancers can serve as a predictive of cell fate for progenitor cells. To achieve this aim, multiple neural progenitor cells at different embryonic stages are required. Consequently, the availability of pure marker genes for the different intermediate neural cell types during neural stem cells differentiating to multiple mature neural cells is a prerequisite.

ATP-dependent chromatin remodeling enzymes are a key factor regulating nucleosome positioning. Different families of chromatin remodelers impact nucleosome organization in distinct fashions. For example, chromatin remodeler ISWI facilitates nucleosome placement in DNA sequences favoring nucleosome formation whereas remodeler families (P)BAP, NURD, and INO80 help nucleosome placement in DNA sequences disfavoring nucleosome formation^34^. Knockdown of Brhama complex, the ATPase subunit of SWI/SNF class of chromatin remodelers, shows that Brahma regulates multiple physical properties of *in vivo* nucleosome positioning during *Drosophila* embryonic development^35^. Which chromatin remodeling enzyme(s) are involve in NDR formation within enhancers during the glial differentiation? Further screening, *Drosophila* line generation, knockdown etc. assays are needed to address this question. This will largely improve our understanding of *in vivo* chromatin remodeling and its role during the glial differentiation.

## Methods

### *Drosophila* lines

The transgenic line *w*^*1118*^; *p[UASRG]6* (III)^17^ was a gift from Professor Steven Henikoff, Fred Hutchinson Cancer Research Center. This stock expresses the transgene *3xFLAG-BLRP-mCherry-RanGap* and *BirA* under the control of GAL4. Other *Drosophila* lines *Gcm-Gal4, UAS-mCDGFP* (II), *Repo-Gal4/Tm3* (III), *Sp/Cyo; Dr/Tm6B* (II, III), and *Tm3/Tm6B* (III) were obtained from the Bloomington stock center.

The *Drosophila* line used to collect *Gcm*-expressed neural progenitor nuclei is generated as follows: G*cm-Gal4, UAS-mCDGFP* virgin flies were crossed with *Sp/Cyo; Dr/Tm6B* male flies to generate F1 offspring *Gcm-Gal4, UAS-mCDGFP/Cyo; +/Tm6B* (female). *w*^*1118*^; *p[UASRG]6* virgin flies were crossed with *Sp/Cyo;Dr/Tm6B* male flies to generate F1 offspring *+/Cyo*; *p[UASRG]6/Tm6B* (female). These two types of F1 female virgins were crossed with *Sp/Cyo; Dr/Tm6B* male flies to create F2 offspring *Gcm-Gal4, UAS-mCDGFP/Cyo; Dr/Tm6B* (female) and *Sp/Cyo*; *p[UASRG]6/Tm6B* (male), respectively. The F2 fly cross produces F3 offspring *Gcm-Gal4, UAS-mCDGFP/Cyo; p[UASRG]6/Tm6B*. The selfcross of F3 flies generates F4 offspring *Gcm-Gal4, UAS-mCDGFP; p[UASRG]6*. The embryos of F4 flies by selfcross were collected to isolate the neural progenitor cells.

The *Drosophila* line used to collect glial nuclei is generated as follows: *Repo-Gal4/Tm3, Ser, Twi-GFP* virgin flies were crossed with *w*^*1118*^*; p[UASRG]6* male flies. The male F1 offspring (*Repo-Gal4*, *p[UASRG]6/Tm3*) was retained by checking mcherry fluorescence in the third larva by fluorescence microscope, and crossed with *Tm3/TM6B* virgin flies to generate F2 offspring *Repo-Gal4*, *p[UASRG]6/Tm3* flies. The embryos of F2 flies by selfcross were collected to isolate the glial cells.

### Antibodies

Antibodies for ChIP assays include H3K4me1 (ab8895, Abcam), H3K4me3 (ab8580, Abcam), H3K27ac(ab4729, Abcam), H3K27me3 (ab6002, Abcam), H3K9ac (ab10812, Abcam). Antibodies for immunostaining are mouse-Repo from Developmental Studies Hybridoma Bank (DSHB), donkey anti-mouse IgG H&L (Cy™5, Jackson ImmunoResearch). Anti-Flag-coated M2 magnetic beads (Sigma-Aldrich) were used for affinity purification.

### Collection of *Drosophila* embryos

Embryos were collected on grape juice plates with yeast paste from embryo collection cages for 2 hr, and allowed to develop for 5 and 12 additional hours at 25 °C. Then embryos are 5–7h-old and 12–14h-old, respectively and collected for cross-linking. Embryos were transfered onto the mesh with PBST (PBS (137 mM NaCl, 4.3 mM Na_2_HPO_4_, 1.4 mM NaH_2_PO_4_) + 0.1% Triton-X-100), and were rinsed with tap water to remove the yeast. Then embryos were dechorionated with 50% solution of bleach for 3 minutes and were cross-linked in a 1:3 mix of ChIP-fixed buffer (50 mM pH 7.6 HEPES, 100 mM NaCl, 0.1 mM EDTA, 0.5 mM EGTA) with 1.8% formaldehyde and heptane for 15 min on a shaker with speed at 300 rpm. The aqueous and organic phase was replaced PBST with 0.25 mM glycine to terminate cross-linked reaction, and fixed embryos were rinsed by PBST for 3 times, and were store at −80 °C.

### Purification of affinity-tagged nuclei from *Drosophila* embryos

Purification of tagged nuclei was performed using INTACT technology as described previously^17^,^36^. Briefly, 0.3–0.5 g fixed embryos were suspended in 4 mL of cold HB125 buffer (15 mM NaCl, 40 mM KCl, 15 mM pH 7.5 Tris-HCl, 0.125 M sucrose, 0.5 mM spermidine, 0.15 mM spermine, EDTA, 0.5 mM EGTA, 1 X Complete protease inhibitor (PI)) and dounce homogenized. Add 0.5 mL of 5 mg/mL DAPI solution if it is needed to monitor bead binding. Nuclei mixture was filtered through one layer of Miracloth into 50 mL conical tube and diluted to 40 mL of cold HB125 buffer, and added 3 mL of Optiprep (Sigma-Aldrich), then centrifuged at 1000 g for 10 min at 4 °C. The supernatant and Optiprep cushion were discarded, leaving ~2 mL of solution containing nuclei concentrated at the interface. Isolated nuclei were suspended in HB125 with 60 μL of anti-Flag M2 magnetic beads slurry and incubated on a rotator for 2 hr at 4 °C. Beads with affinity-bound nuclei were absorbed by magnet and washed by HB125 three times. Purified nuclei are stored at −80 °C for future use.

### MNase-seq and ChIP-seq

Affinity-purified nuclei were collected by centrifugation at 1000 g for 5 min, suspended in 500 μL 37 °C pre-heated MNase digestion buffer (10 mM pH 7.5 Tris–HCl, 15 mM NaCl, 60 mM KCl, 2 mM CaCl_2_, 0.15 mM spermine, 0.5 mM spermidine, 1X PI) with 12U MNase (Micrococcal nuclease, Worthington Biochemical Corporation), and then incubated at 37 °C for 20 min. Terminated the digestion on ice for 10 min by adding EDTA to a final concentration of 10 mM. Supernatant was discarded and pellet was washed with A2 buffer (140 mM NaCl, 15 mM pH 7.6 HEPES, 1 mM EDTA, 0.5 mM EGTA, 0.1% Triton X-100, 0.1% sodium deoxycholate, 1X PI), and resuspended in A2 buffer with 0.1% SDS. The pellet was dissolved through sonication with 3 cycles of 20 s duration with at least 40 s pause between cycles at the power setting of 6 (out of 20) on Misonix sonicator XL-2000. The supernatant was retained for next ChIP assay, or reversal of cross-linking to harvest nucleosomal DNA as follow: the supernatant was treated with RNase A at 37 °C for 0.5–1 hours and Proteinase K at 65 °C for 2 hours, respectively. Next, the nucleosomal DNA mixture was extracted by phenol-chloroform and precipitated with a 1:10:100:200 mix of 20 mg/mL glycogen, 3 M pH 5.3 NaOAc, nucleosomal DNA mixture and cold 100% ethanol.

We used 10–15 μg of chromatin for each chromatin immunoprecipitation (IP) reaction with histone modification antibodies of appropriate doses as indicated in the specification. Mixture contained chromatin, antibody, and ChIP buffer (16.7 mM pH 8.1 Tris-HCl, 167 mM NaCl, 1.2 mM EDTA, 1% Triton X-100, 0.01% SDS) were incubated on a rotator overnight at 4 °C. Then 20 μL of ChIP Grade Protein G Magnetic Beads (Cell Signaling #9006) was added to each IP reaction. The mixture was incubated for 2 hr with rotation. Next, beads were washed three times with low salt wash buffer (2 mM EDTA; 20 mM pH 8.1 Tris-HCl, 0.1% SDS, 1% Triton X-100, 150 mM NaCl) and once with high salt wash buffer (2 mM EDTA, 20 mM pH 8.1 Tris-HCl, 0.1% SDS, 1% Triton X-100, 500 mM NaCl), 5 min for each wash. Beads were suspended in 150 μL of ChIP elution buffer (50 mM pH 8.1 Tris-HCl, 10 mM EDTA, 0.1% SDS) at 65 °C for 45 min. Finally, we reversed cross-linking to harvest ChIP’ed DNA fragments and sequenced them on Illumina HiSeq2000 platform using 49 bp single end protocol.

### Immunofluorescence staining

We dechorionated embryos in 50% solution of bleach and then fixed them in a 1:1 mix of 4% formaldehyde in PBS with 0.3% Tween 20 and heptane for 20 min on a shaker. The aqueous phase was discarded and replaced with methanol, and embryos were shaken for 3~5 min at 300 rpm to burst vitelline membranes. Embryos were rinsed three times in methanol and rinsed three times in PBS with 0.3% triton X-100. Embryos were blocked with PBST supplemented by 5% normal donkey serum (NDS) for 30 min, then incubated with primary antibodies of various dilutions in PBST containing 5% NDS overnight at 4 °C. The embryos were washed as above described, followed by incubation with the secondary antibody for 1–2 hours, then were washed three times again to avoid non-specific binding. The stained embryos were mounted on slides in PBS with additional 50% glycerol. Slides were examined on Zeiss Imager M2 microscopy. To evaluate the purity of affinity-isolated nuclei, DAPI-stained total and affinity-purified nuclei were counted on a hemacytometer, respectively.

### RNA-seq analysis

Nuclear RNA was extracted from affinity-purified tagged nuclei using the RNeasy Micro Kit (Qiagen). Genomic DNA was removed with Turbo DNA-free kit (Ambion). The cDNA library for RNA sequencing was constructed using standard Illumina libraries prep protocols. Sequencing was conducted on Illumina HiSeq2000 platform using 49 bp single end protocol. Sequencing reads were mapped to *Drosophila* transcripts (FlyBase r5.43) using Tophat (v1.3.1) with default parameters setting^37^. Uniquely mapped reads were assembled into transcripts guided by reference annotation with Cuffdiff (v1.3.0)^37^ to calculate gene expression levels that were normalized as Read Per Kilobase per Million mapped reads (RPKM). The differentially expressed genes were identified with FDR < 0.05.

### Functional annotation analysis

Gene ontology analysis of gene set was performed using the functional annotation tool DAVID (v6.7)^38^.

### Histone modification change in the promoters

Histone modification sequencing reads were mapped to the *Drosophila* reference genome (dm3) with up to two mismatches using Bowtie^39^. Promoters are defined as the ±1 kb regions of the TSSs. Each read represents a modified nucleosome and was extended toward 3′ end to a length of 147 bp. The midpoint of the extended read defines the read position. Read distance to TSS were binned in 5-bp interval. Histone modification signal is measured by the read count in the bins. Finally, histone modification signal in each bin was further normalized as Read Per Million mapped reads (RPM) and smoothed with 5 bins. We further clustering each histone modification signals in promoters by K-means (K = 2) to classify promoters into two groups with or without this histone modification.

### Determination of enhancers and the chromatin states

Enhancers were H3K4me1 enriched peaks that were identified by the tool HOMER using a 1000-bp sliding window with a false discovery rate of 0.1%^40^. The enhancers of the two cell types with distance less than 500 bp were merged by retaining the one with highest HOMER score. Next, we discarded the enhancers at least 50% of whose length overlaps with promoter regions. Then, we calculated the read count of histone modifications (H3K4me1, H3K27ac, H3K27me3) in the ±3 kb regions of enhancer center in 50-bp window (total 120 windows). We further normalized the histone modification occupancy using the total reads within the ±3 kb regions. We next determined the chromatin state of enhancers by clustering the histone modification signals: H3K4me1 + /H3K27ac + as active, H3K4me1 + /H3K27me3- as poised, and the rest as intermediate.

### Prediction of nucleosome positions and analysis of positioning dynamics

Nucleosomal DNA sequencing reads were aligned to the *Drosophila* reference genome (dm3) with up to two mismatches using Bowtie^39^. The uniquely mapped reads were used to predict nucleosome positions using the peak-calling tool GeneTrack^22^. The read counts within each nucleosome calculated by GeneTrack were further normalized as RPM by total uniquely mapped reads. Each nucleosome was assigned to promoter, genic and intergenic regions depending on which region the dyad of nucleosome located.

Nucleosome organization within a region was shown in heatmap as follows: we located nucleosomal reads in the genome that comprise nucleosomes predicted by GeneTrack. Then we summed read counts nucleosome occupancy using a 5-bp bin and smoothed with 5 bins. We further normalized the nucleosome occupancy using the total reads within the region.

## Additional Information

**Accession codes:** The RNA-seq, MNase-seq and ChIP-seq data sets have been deposited in the Gene Expression Omnibus (GEO) under accession number GSE83377.

**How to cite this article**: Ye, Y. *et al.* Chromatin remodeling during the *in vivo* glial differentiation in early *Drosophila* embryos. *Sci. Rep.* **6**, 33422; doi: 10.1038/srep33422 (2016).

## Supplementary Material

Supplementary Information
