# Supplementary material for: Chromatin remodeling during the in vivo glial differentiation in early Drosophila embryos
Source: Sci Rep. 2016 Sep 16;6:33422. doi: 10.1038/srep33422 (PMC5025732; doi:10.1038/srep33422)
Supplement: Supplementary Information [file srep33422-s1.pdf]

Chromatin remodeling during the *in vivo* glial differentiation in early *Drosophila* embryos

Youqiong Ye, Liang Gu, Xiaolong Chen, Jiejun Shi, Xiaobai Zhang* and Cizhong Jiang*

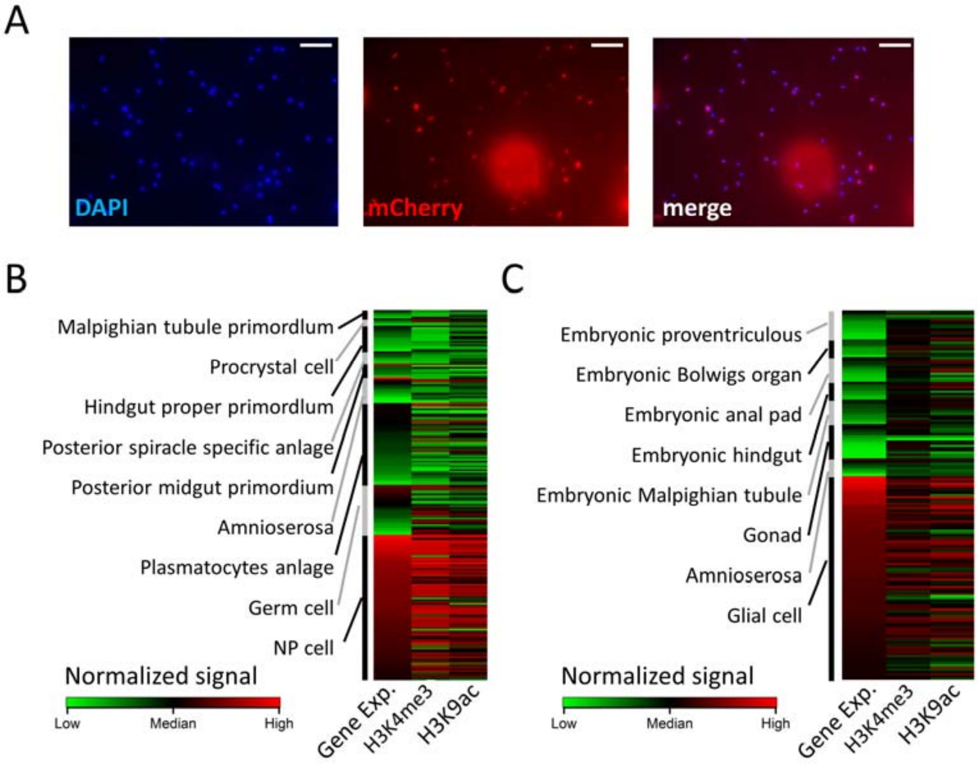

Figure S1. Purity and histone modification profiles of isolated cell type-specific nuclei from *Drosophila* embryos.

(A) High purity of affinity-captured cell type-specific nuclei. The purity of the affinity-purified nuclei is measured by the ratio of the mCherry-positive nuclei count to the total nuclei count. Scale bar: 40 µm. **(B-C)** Heatmaps show the profiles of core histone modifications in the promoter regions (±1 kb of TSS) of different tissue-specific genes in the purified nuclei of GNP (B) and glial (C) cells. Genes are sorted descendingly by expression level within each tissue.

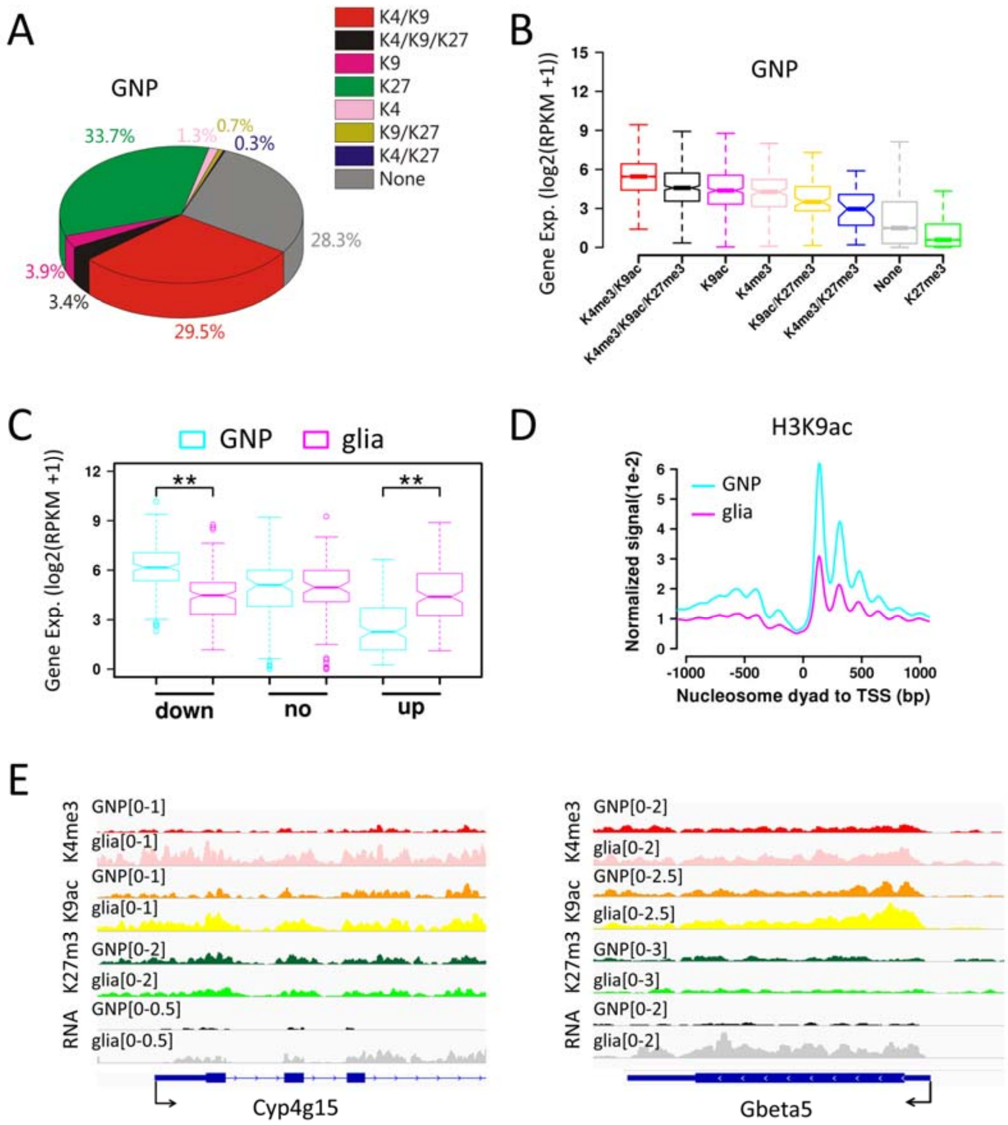

Figure S2. Relationship between gene expression and chromatin state in promoter regions.

(A) Categorization of promoters in the GNP cells by combination of histone modification signals. Similar results are obtained in the glial cells (data not shown here). **(B)** Expression profiles of gene sets grouped by chromatin states in (A). **(C)** Expression profiles of glia-related gene sets grouped by expression change fold in Figure 3A. **(D)** The average H3K9ac signals in the regions around TSS of all genes. **(E)** Track view for expression levels, core histone modification signals in the glial genes *Cyp4g15* and *Gbeta5* in the two cell types.

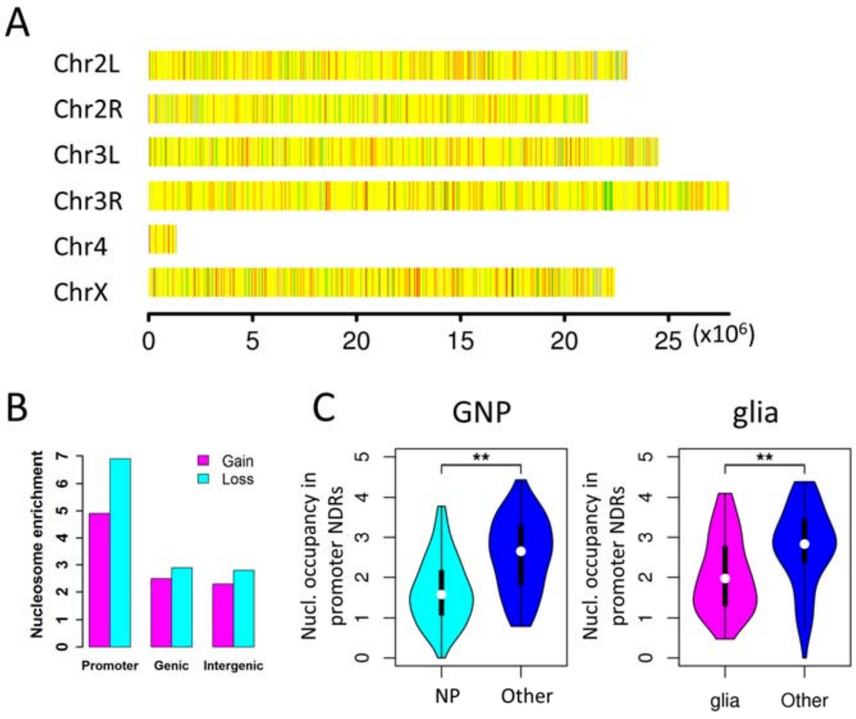

Figure S3. Dynamic nucleosome positioning.
(A) Nucleosome occupancy change across the genome by chromosomes. The ratio of nucleosome occupancy in each 200-bp window is shown in colors. Red regions indicate that nucleosome occupancy in glia is at least 1.5-fold higher than GNP cells. Green regions indicate that nucleosome occupancy in neurons is at least 1.5-fold lower than GNP cells. Grey indicates regions of Ns in the genome. Yellow regions are the rest of regions. **(B)** Enrichment of nucleosome gain and loss in different genomic regions. The enrichment of gained / lost nucleosomes in promoters equals to the number of nucleosomes locating in promoters normalized by the length of promoters. The enrichment of gained / lost nucleosomes in genic and intergenic regions are calculated in the same way. **(C)** Profiles of nucleosome occupancy in the promoter NDRs in NP- and other tissue-specific genes in the GNP cells (left), and in glia- and other tissue-specific genes in the glial cells (right) (**: $p < 0.01$, Wilcoxon rank sum test). Other tissues include all the non-NP tissues and non-glia tissues in Figure S1B, respectively.

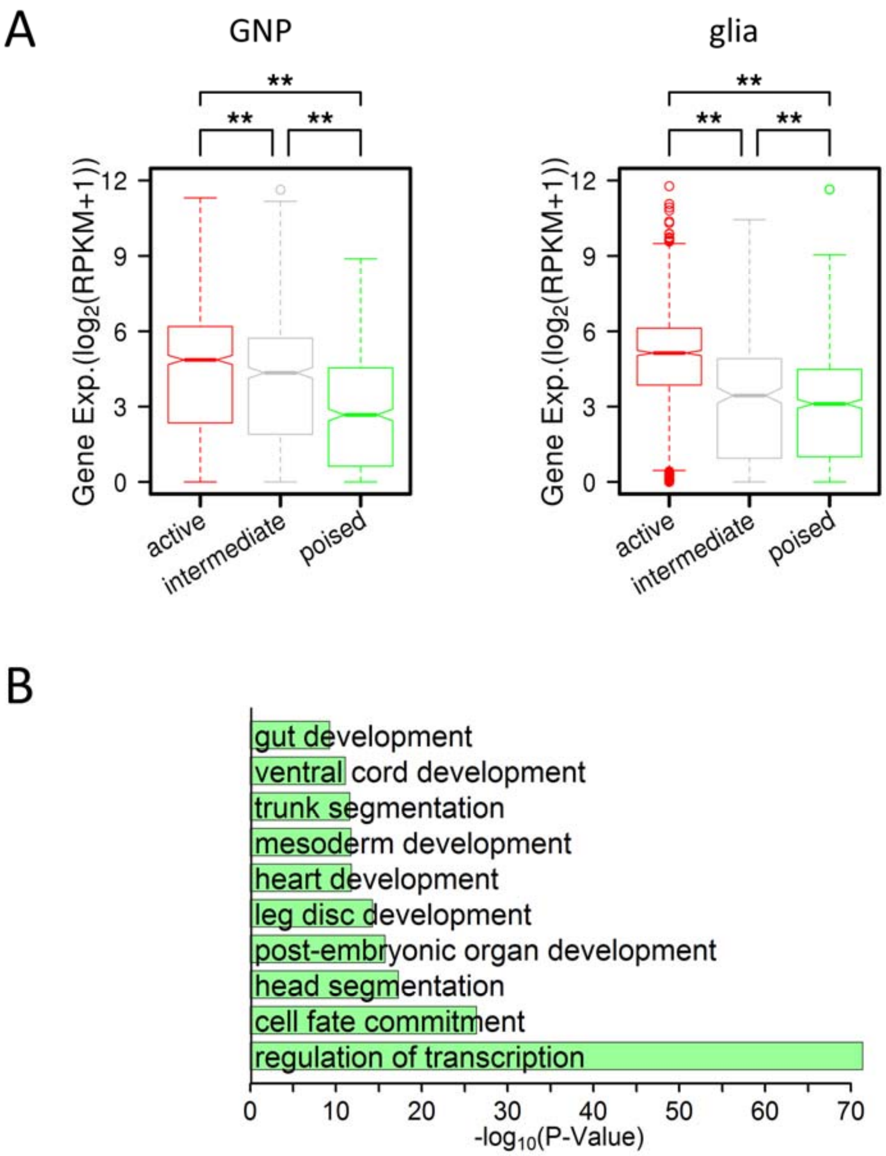

Figure S4. Relationship between chromatin state of enhancers and expression levels of the associated genes.
(A) Expression profiles of the three groups of genes associated with active, intermediate, and poised enhancers, respectively (**: $p < 0.01$, Wilcoxon rank sum test). **(B)** Significantly enriched GO terms for the genes associated with the enhancers remaining poised state during the glial differentiation.
